# Switching from Biosimilar to Biosimilar Adalimumab, Including Multiple Switching, in Crohn’s Disease: A Prospective Study

**DOI:** 10.3390/jcm10153387

**Published:** 2021-07-30

**Authors:** Davide Giuseppe Ribaldone, Elisa Tribocco, Chiara Rosso, Angelo Armandi, Marta Vernero, Elisabetta Bugianesi, Marco Astegiano, Giorgio Maria Saracco, Gian Paolo Caviglia

**Affiliations:** 1 Department of Medical Sciences, Division of Gastroenterology, University of Torino, 10126 Torino, Italy; elisa.tribocco@edu.unito.it (E.T.); angelo.armandi@unito.it (A.A.); elisabetta.bugianesi@unito.it (E.B.); giorgiomaria.saracco@unito.it (G.M.S.); gianpaolo.caviglia@unito.it (G.P.C.); 2 Department of Internal Medicine, San Matteo Hospital, 27100 Pavia, Italy; martavernero@gmail.com; 3 Department of General and Specialist Medicine, Gastroenterologia-U, Città della Salute e della Scienza di Torino, C.so Bramante 88, 10126 Turin, Italy; mastegiano@cittadellasalute.to.it

**Keywords:** originator, Amgevita, Amjevita, Imraldi

## Abstract

No data are available regarding the safety and effectiveness of the biosimilar-to-biosimilar switch of adalimumab in any disease, and in particular in Crohn’s disease (CD). The aim of our study was to provide real world data on switching from biosimilar adalimumab to another biosimilar, including multiple switching. We conducted a prospective, single-centre observational study in which we consecutively recruited all CD patients who switched from adalimumab biosimilar ABP 501 to biosimilar SB5 from January to July 2021. Sixty-one patients were included in the final analysis, of whom 43/61 (70.5%) were multiple switches (Humira^®^ → ABP 501 → SB5). After 6 months of follow up, 88.5% (54/61) of patients maintained SB5 on therapy. The success of the switch (defined as no systemic corticosteroids within 6 months, non-discontinuation of SB5, no dose escalation) was achieved by 82.0% (50/61) of patients. At multivariate analysis, C-reactive protein > 5 mg/L predicted switch failure (*p* = 0.03). Seven patients (11.5%) experienced side effects, compared to one patient (1.6%) in the 6 pre-switch months (*p* = 0.03). In conclusion, switching from biosimilar to biosimilar of adalimumab did not lead to signs of safety or loss of efficacy other than those already known in the literature for the class of drugs.

## 1. Introduction

Crohn’s disease (CD) is a chronic, life-long disease [1]. The introduction of biological therapy in the treatment of CD has led to great benefits. In particular, there was an important improvement from the point of view of symptoms, of the inflammatory state, and of the quality of life [2]. However, one of the problems with biological therapy is the high cost, which prevents it from being prescribed to a larger number of patients.

Adalimumab (originator Humira^®^, Abbvie, North Chicago, IL, USA), introduced in 2007, is a fully human IgG1 monoclonal antibody with subcutaneous administration directed against tumor necrosis factor (TNF) [3].

Biosimilars are biological products similar, but not identical, to an already approved biological drug, called “originator”: this is due to the complex production process [4]. Compared to the originator, the biosimilar drug must demonstrate that it is not clinically different in terms of efficacy and safety [5]. According to the European Medicines Agency (EMA), if it is established that the biosimilar drug is highly similar to the originator, with a similar safety and efficacy profile in a therapeutic indication, the safety and efficacy data can be extrapolated to other indications authorized for the originator [6]. Although it is recognized that extrapolation allows the development of biosimilars to expand, the medical community discusses several issues, especially with regard to the application of a biosimilar to a therapeutic indication for which there are no data from clinical trials [7,8].

Currently, biosimilars are used either in drug-naïve patients or as a switch. The switch typically occurs between originator and biosimilar; however, in clinical practice, with increasingly new biosimilars on the market, the switch from biosimilar to biosimilar is more and more frequent. Regarding infliximab, in a recent prospective study, the switch from biosimilar CT-P13 to biosimilar SB2 was evaluated in patients with inflammatory bowel disease: the results showed that the efficacy and safety of the therapy remained stable for the duration of the follow-up [9]. Regarding adalimumab biosimilars, the first introduced was ABP 501 (Amgevita^®^, Amgen, Thousand Oaks, CA, USA). A recent observational study, conducted on a population of 87 patients with CD treated with ABP 501, demonstrated a clinical benefit comparable to that given by originator therapy [10]. SB5 (Imraldi^®^, Biogen, Denmark—Samsung Bioepis, Incheon, South Korea) was the second adalimumab originator biosimilar to be approved after ABP 501. Also in this case, there is little evidence on the use of this drug for the treatment of inflammatory bowel diseases. Recently, a study evaluated the response to SB5 in a population of patients with CD and ulcerative colitis who switched from adalimumab originator to SB5, reporting the same clinical activity 6 months after the switch [11].

It is evident that, in the context of inflammatory bowel diseases, there is still little evidence available regarding the switch from originator to biosimilar and even less data regarding the switch between biosimilars, in particular with regard to adalimumab [12]. Based on these premises, our study aimed to evaluate the effectiveness and safety of the switch from biosimilar ABP 501 (Amgevita^®^) to biosimilar SB5 (Imraldi^®^) in patients with CD on maintenance therapy with adalimumab. At present, there are no similar studies in the literature.

## 2. Materials and Methods

We conducted a prospective, single-centre, observational study. Patients were included in the “IBD cohort” (Ethics Committee Approval No. 0056924).

### 2.1. Included Patients

In the period between January 2020 and July 2020, all patients with CD who switched, in accordance with regional indications, from the biosimilar of adalimumab ABP 501 to SB5 (T0) were consecutively recruited. 

The inclusion criteria were:

- CD diagnosed according to ECCO criteria [13].
- Age ≥ 16 years.
- Maintenance therapy with ABP 501 for at least 8 months.

The exclusion criterion was:

- Switch to another adalimumab biosimilar (not SB5).

### 2.2. Data Collection

Patients underwent a visit at T0. The following parameters were collected:

- Baseline characteristics: age, gender, smoking habit.
- Clinical history: disease duration, localization of CD according to the Montreal classification [14], history of perianal disease, CD-related hospitalization, CD-related intestinal surgery.
- Clinical activity, assessed with the Harvey–Bradshaw Index (HBI) [15].
- Laboratory activity: C-reactive protein (CRP, mg/L) and faecal calprotectin (mg/kg).
- Previous and current medical therapy: previous treatment with adalimumab originator (multiple switch), history of treatment with infliximab, concomitant therapy (corticosteroids and/or thiopurines), dose escalation of ABP 501 and SB5, frequency of administration of ABP 501 and SB5, adverse events to ABP 501 and SB5.

### 2.3. Outcomes

The primary outcome of the study was to determine the success of the switch to SB5, defined as: no systemic corticosteroids within 6 months (T6) of therapy with SB5, non-discontinuation of SB5 during the period between the initiation of therapy with SB5 (T0) and the sixth month of observation (T6), no dose escalation in the period between T0 and T6. The secondary outcomes were: comparison of the last 6 months of ABP 501 therapy and the first 6 months of SB5 therapy regarding dose escalation rate, use of systemic corticosteroids, use of thiopurines, CRP, faecal calprotectin, HBI. Univariate and multivariate analyses were performed to look for predictor of successful switch to SB5. All adverse events were collected.

### 2.4. Statistical Analysis

Categorical variables were expressed as number and percentage (%). Regarding the continuous variables, the D’Agostino–Pearson test was applied to evaluate if they were normally distributed. The normally distributed variables were expressed as mean ± standard deviation (SD) (or as geometric mean if they were normally distributed after logarithm transformation), those not normally distributed were expressed as median and interquartile range (IQR). The chi-square test was used to compare the trend of categorical variables pre- and post-switch. For the comparison of the trend of continuous variables not normally distributed, the Friedman test was used. For the comparison of the trend of continuous variables normally distributed, the ANOVA (analysis of variance for repeated measures) test was applied. For the comparison of paired continuous variables, the Wilcoxon test was applied. The multivariate analysis was performed using logistic regression. The results of all analyses were considered statistically significant for *p* < 0.05. Statistical analysis was performed with MedCalc Statistical Software version 18.9.1 (MedCalc Software bvba, Ostend, Belgium; http://www.medcalc.org; 2018; accessed on 15 May 2021).

## 3. Results

In total, 68 patients were recruited, including 6 lost to follow-up and 1 excluded due to switching to another biosimilar of adalimumab originator (MSB11022) (Table 1).

### 3.1. Efficacy

At the end of the follow-up, the success rate was 82.0% (50 out of 61 patients). Regarding failure (11 patients, 18.0%), 7 patients discontinued SB5 and 4 patients, of whom 2 received systemic corticosteroids, increased the frequency of SB5 administration. The maintenance rate of SB5 therapy at 6 months was 88.5% (54/61). The causes of SB5 discontinuation were the presence of adverse events that required swap to another drug in 2 patients, and the failure in controlling the disease in five patients. Of the 2 patients who experienced adverse effects, 1 swapped to vedolizumab, the other switched to the adalimumab biosimilar GP2017. Of those who experienced drug failure, 2 swapped to ustekinumab and 3 underwent surgical resection.

In the last 6 months of treatment with ABP 501 prior to the switch, 3.3% (2/61) of patients underwent dose escalation, while in the 6 months following the switch to SB5, there was a dose escalation rate of 6.6% (4/61) (difference not statistically significant, *p* = 0.44). No differences were reported regarding the use of systemic corticosteroids between the period with ABP 501 and that with SB5 (rate of 3.3% for both, *p* = 1.0). As regards thiopurines use, at the time of the switch there was a rate of 8.2% (5/61), while after 6 months of therapy with SB5 the rate was 4.9% (3/61) (*p* = 0.48). 

### 3.2. Biochemical and Clinical Parameters Trend

The mean CRP value 6 months before switch (T-6) and at the time of switch (T0) was 1.5 mg/L (95% confidence interval (CI) 1.0–2.3 mg/L) and 1.3 mg/L, respectively (95% CI 0.8–1.9 mg/L), not significantly different from that reported after six months of SB5 therapy (1.3 mg/L, 95% CI 0.9–2.0 mg/L, *p* = 0.6). The mean faecal calprotectin value at T-6 and T0 was 121.6 mg/kg (95% CI 67.2–220.1 mg/kg) and 83.8 mg/kg (95% CI 47.6–147.6 mg/kg), respectively, not significantly differing from that reported after 6 months of SB5 therapy (86.3 mg/kg, 95% CI 49.2–151.3 mg/kg, *p* = 0.4). The median value of HBI at T-6 and T0 was 2.0 (95% CI 0.0–4.0) and 1.0 (95% CI 1.0–4.0), respectively, while after 6 months of therapy with SB5 the median value was 2.0 (95% CI 1.0–3.5, *p* = 0.5).

### 3.3. Predictors of Success of the Switch to SB5

#### Univariate Analysis

Patient and disease characteristics were evaluated as possible predictors of switch success (Table 2).

**Figure 1:**
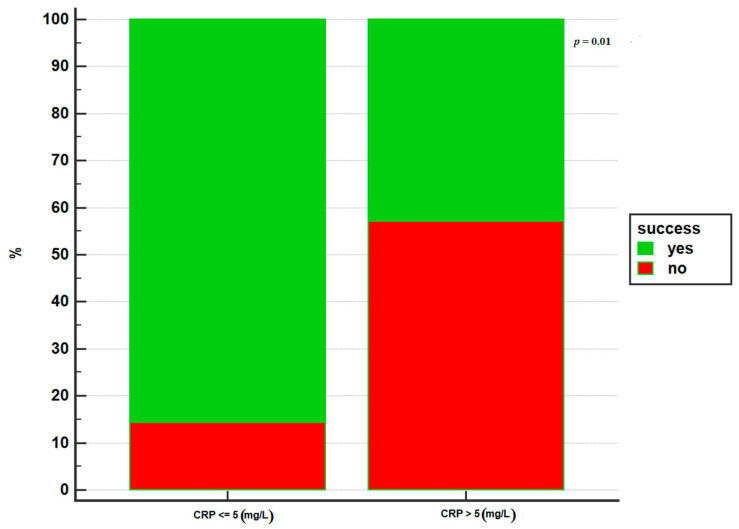
CRP value at the switch and success of the switch.

The only parameter that was associated with failure was a positive PCR at the time of the switch: a CRP > 5 mg/L was associated with a lower success of the switch, with an OR = 0.13 (95% CI 0.02–0.70, *p* = 0.02) (Figure 1).

### 3.4. Multivariate Analysis

At the multivariate analysis, the predictors of switch success of greatest clinical interest were simultaneously evaluated (Table 3). 

Multiple switches, a positive CRP, and a positive calprotectin (>250 mg/kg) were analysed: also in this case, a positive CRP was statistically significantly associated (*p* = 0.03) with lower success of the switch (OR = 0.07, 95% CI 0.01–0.74).

### 3.5. Adverse Events

In the last 6 months of therapy with ABP 501, one patient developed one adverse event (1.6%): the patient developed paradoxical psoriasis. In the 6 months following the switch to SB5, adverse events occurred in seven patients (11.5%). Of these, two occurred immediately or a few hours after drug administration (one patient reported asthenia and one reported pain at the site of administration). In one patient there was an onset of peripheral arthritis. Dermatological adverse events were reported in three patients, one of which was characterized by itching and wheals at the injection site, one by the onset of paradoxical psoriasis, and the other by worsening of acne. Finally, one patient reported abdominal pain. Comparing the rate of adverse events in the 6 pre-switch and in the 6 post-switch months the difference was statistically significant (*p* = 0.03).

## 4. Discussion

Currently, the guidelines recommend not switching between biosimilars and multiple switches until there are clinical data to support this practice [4,16]. To date, no data are available in the literature on the safety and efficacy of multiple switches and, in particular, on switching from adalimumab biosimilar to biosimilar.

In light of these considerations, we conducted a prospective study that, for the first time, evaluated the effectiveness and safety of switching from the adalimumab biosimilar ABP 501 to the biosimilar SB5 in a population of 61 patients affected by CD in maintenance therapy with ABP 501, comparing the 6 months before and the 6 months after the switch. At the end of the follow-up, a switch success rate (defined as no systemic corticosteroids within 6 months of therapy with SB5, non-discontinuation of SB5, and no dose escalation) of 82.0% (50/61) was found. Regarding loss of response (11 patients, 18.0%), 7 discontinued SB5, 4 (of whom 2 received systemic corticosteroids) increased the frequency of administration (dose escalation). In the literature, the loss of response to adalimumab originator at 12 months is between 23–46% [17], comparable to that found in our study. Equally remarkable was the SB5 retention rate, which, at the end of the follow-up, was 88.5% (54/61 patients). The causes of drug stop were the loss of response in 5/61 (8.2%) patients and an adverse event in 2/61 (3.3%) patients. SB5 stop rate after 6 months (11.5%) was comparable to that of adalimumab originator (up to 13% at 1 year) [17]. 

Comparing the 6 months of treatment with ABP 501 prior to the switch with the 6 months of therapy with SB5 after the switch, there was no significant increase in dose escalation rate (*p* = 0.4). Furthermore, the number of patients receiving oral corticosteroids remained stable (3.3%), while there was a slight decrease in patients in combination therapy with thiopurines (from 8.2% to 4.9%, not significant *p* = 0.5). 

Regarding the inflammation markers, the CRP and faecal calprotectin values at the end of the follow-up did not increase significantly (*p* = 0.6 and *p* = 0.4, respectively) compared to the values at the time of switching. Even clinically, after 6 months of SB5 therapy, no significant increase in the HBI value (*p* = 0.5) was reported, confirming a stable course of the disease after the switch. 

Our findings seem to agree with those found in a recent French prospective study, which evaluated the efficacy and safety of switching of infliximab biosimilar (CT-P13 to SB2) in a cohort of 158 patients with inflammatory bowel disease, including 110 patients with CD [9]. After 54 weeks of follow-up, an SB2 maintenance rate of 94.9% was reported; 9.8% of patients reported a loss of response to SB2. Another study that evaluated the efficacy and safety of switching between infliximab biosimilars (from CT-P13 to SB2) and of multiple switches in a population with inflammatory bowel disease, followed for 48 weeks, is the study conducted by Macaluso et al. [18]; 43 patients underwent the switch from CT-P13 to SB2 and 24 patients underwent multiple switches. Drug retention at 48 weeks was 75.3% and 82.4%, respectively. The adverse event rate was 25.6% in the first group and 16.7% in the multiple switch group. 

By evaluating the predictive factors of switch success, it was found that having a positive CPR at the time of the switch correlated with the failure of the switch (both in the univariate analysis and in the multivariate, *p* = 0.02 and *p* = 0.03, respectively). This confirms that an elevated CRP is a prognostic factor for non-maintenance of therapy, as has been found with adalimumab originator [19]. A datum of great clinical relevance is represented by the fact that the multiple switches did not affect the success of the switch (*p* = 0.91), confirming the data concerning infliximab by Macaluso et al. [18]. 

Regarding the safety profile, the switch from ABP 501 to SB5 did not cause a significant increase in either the rate of CD-related hospitalization or the rate of surgery (*p* = 0.2 and *p* = 0.16, respectively). As regards the adverse events, however, they occurred in 11.5% of patients, with a statistically significant increase compared to the last 6 months of therapy with ABP 501 (*p* = 0.03). This increase could be partly explained by a mechanism called the “nocebo effect”, a phenomenon observed in patients with inflammatory bowel disease who underwent a switch from the originator drug to a biosimilar drug [20]. This is especially true if we consider that patients had become “accustomed” to ABP 501 therapy, and we analysed the first 6 months after the switch. Evaluating the specific adverse events found in our study, the relative majority were dermatological (3/7 42.9%). There were no life-threatening safety events, including malignant diseases and tuberculosis infections, during the observation period. These data seem to confirm, at least in the short term, that the switch from biosimilar to biosimilar was generally well tolerated and has a good safety profile. It should be emphasized that the moment of the switch is particularly delicate. Therefore, it is important to set up a communication strategy, based on the data in the literature, which reassures the patient and provides the correct information on the new drug, in order to reduce the impact of the nocebo effect.

A potential limitation of this study was the relatively small sample size, which may have limited the generalization of the results; on the other hand, ours is the first study regarding multiple switches of adalimumab in any of the diseases for which it is authorized. Another criticism of our study is the lack of endoscopic outcome, but this depend on the fact that this study is a real world study and only a small minority of the patients, who were on maintenance with adalimumab therapy, underwent this examination during follow-up. Furthermore, we evaluated an endoscopy surrogate marker like calprotectin [21], which confirmed the effectiveness of the switch. 

## 5. Conclusions

In conclusion, our study suggests that switching (including multiple switches) from adalimumab biosimilar to biosimilar in patients with CD is safe and effective. It is not recommended to switch if positive CRP is found at the time of switching. These results should encourage to perform larger studies with longer follow-up to obtain further data on the efficacy and safety of this practice.

## Figures and Tables

**Table 1:** Baseline demographic characteristics of patients undergoing switch from ABP 501 to SB5 (*n* = 61).

Baseline Characteristics of the Patients	
Age at time of switch with SB5 (year), median (IQR)	48 (34.3–60.5)
Smoking (never/active/former)	20/19/22
Gender (male/female)	45/16
Harvey–Bradshaw Index (HBI), median (IQR)	1 (1–4)
C-reactive protein (mg/L), geometric mean (95% CI)	1.3 (0.8–1.9)
Faecal calprotectin (g/kg), geometric mean (95% CI)	19.7 ± 12.3
Previous adalimumab originator (multiple switch), yes/no	43/18
ABP 501 months at the time of the switch, mean ± SD	13.5 ± 3.1
Adalimumab months at switch, median (IQR)	27 (14.8–57.3)

CI = confidence interval; IQR = interquartile range.

**Table 2:** Predictors of success of the switch at univariate analysis.

Parameters	OR (95% CI)	*p* Value
Age	0.97 (0.93–1.02)	0.19
Sex	1.75 (0.34–9.13)	0.51
Localization of disease in the colon	0.86 (0.22–3.31)	0.82
Perianal disease	0.38 (0.10–1.47)	0.16
Disease duration	0.98 (0.93–1.03)	0.43
Hospitalization	NF	
Multiple switch	0.88 (0.20–3.76)	0.86
Adalimumab months	0.999 (0.98–1.02)	0.90
Frequency of adalimumab administration	0.64 (0.06–6.79)	0.71
Combined therapy with thiopurines at T0	NF	
Combined corticosteroid therapy at T0	0.22 (0.01–3.78)	0.29
CRP > 5 mg/L at T0	0.13 (0.02–0.70)	0.02
Faecal calprotectin > 250 mg/kg at T0	1.75 (0.18–16.65)	0.63
HBI at T0	0.82 (0.62–1.07)	0.15

OR = Odds Ratio; CI = confidence interval; CRP = C-reactive protein; HBI = Harvey–Bradshaw Index; NF = not feasible.

**Table 3:** Predictors of success of the switch at multivariate analysis.

Parameters	OR (95% CI)	*p* Value
Multiple switch	1.11 (0.19–6.49)	0.91
CRP > 5 mg/L	0.07 (0.01–0.74)	0.03
Faecal calprotectin > 250 mg/kg	4.64 (0.26–82.16)	0.29

OR = Odds Ratio; CI = confidence interval; CRP = C-reactive protein.

## Data Availability

The data were collected anonymously. Anonymous data can be requested in case of need.
